# Running Loose or Getting Lost: How HIV-1 Counters and Capitalizes on APOBEC3-Induced Mutagenesis through Its Vif Protein

**DOI:** 10.3390/v4113132

**Published:** 2012-11-14

**Authors:** Carsten Münk, Björn-Erik O. Jensen, Jörg Zielonka, Dieter Häussinger, Christel Kamp

**Affiliations:** 1 Clinic for Gastroenterology, Hepatology and Infectiology, Medical Faculty, Heinrich Heine University, 40225 Düsseldorf, Germany; Email: carsten.muenk@med.uni-duesseldorf.de (C.M.); bjoern-erikole.jensen@med.uni-duesseldorf.de (B.-E.O.J.); joerg.zielonka@roche.com (J.Z.); dieter.haeussinger@med.uni-duesseldorf.de (D.H.); 2 Roche Glycart AG, Schlieren 8952, Switzerland; 3 Paul-Ehrlich-Institut, Federal Institute for Vaccines and Biomedicines, Paul-Ehrlich-Straße 51-59, 63225 Langen, Germany

**Keywords:** HIV-1, APOBEC3, Vif, coevolution, quasispecies, population genetics, modeling, drug resistance, escape mutant

## Abstract

Human immunodeficiency virus-1 (HIV-1) dynamics reflect an intricate balance within the viruses’ host. The virus relies on host replication factors, but must escape or counter its host’s antiviral restriction factors. The interaction between the HIV-1 protein Vif and many cellular restriction factors from the APOBEC3 protein family is a prominent example of this evolutionary arms race. The viral infectivity factor (Vif) protein largely neutralizes APOBEC3 proteins, which can induce *in vivo* hypermutations in HIV-1 to the extent of lethal mutagenesis, and ensures the production of viable virus particles. HIV-1 also uses the APOBEC3-Vif interaction to modulate its own mutation rate in harsh or variable environments, and it is a model of adaptation in a coevolutionary setting. Both experimental evidence and the substantiation of the underlying dynamics through coevolutionary models are presented as complementary views of a coevolutionary arms race.

## 1. Introduction

After the early years of the human immunodeficiency virus/acquired immunodeficiency syndrome (HIV/AIDS)-epidemic, monotherapy with the first antiretroviral active drug azidothymidine (AZT, licensed 1987) was enthusiastically embraced [1]. However, due to frequent mutations in the HIV-1 genome and ensuing selection of drug-resistant viral strains, AZT monotherapy failed to improve long-term clinical outcomes. Over the following 25 years, HIV specialists gradually realized that an elaborate combinated antiretroviral therapy (cART) was required to reach a sustained suppression of HIV-1 and to transform the infection from a death sentence into a manageable chronic disease. But even with the successful use of cART since 1995 and the development of phenotypic and genotypic assays for the analysis of antiretroviral drug resistance therapy, failure due to drug resistance remains a major obstacle in the treatment of HIV-infected patients. Despite the existence of more than 25 licensed antiretroviral drugs having six different modes of action, the demand for new ones is still high, particularly in the light of the longer life expectancy and, therefore, the extended duration of required antiretroviral treatment in HIV-infected persons. Another important issue in this context is the transmission of resistant strains of HIV-1 and its potential effect at the population level. Prevalence of primary drug resistance mutations of around 10% was detected in several European cohorts (e.g., the RESINA-study [2]). Major selection pressures on HIV-1 apart from antiretroviral drugs are the human innate and adaptive immune system. HIV-1 manages to escape eradication by drugs and immune responses through a strategy of high turnover, a large viral population, and enormous variation due to its error-prone reverse transcriptase making about one error per 10,000 nucleotides, as well as recombinogenic effects [3,4]. Thus, well-adapted viral populations (or quasi-species) are rapidly selected in each host. Attempts to boost or target immunity against HIV-1 through vaccination efforts show very limited success, and the regular emergence of mutant viruses resistant to administered therapy necessitates the development of new drugs [5,6]. 

It is very possible that genetic polymorphisms in dependency factors (such as the CD4 receptor used for cell entry), in addition to differential immune control, cause the high variability in clinical HIV-1 disease progression. In mechanistic contrast to the dependency factors are the cellular antiviral proteins called restriction factors, some of which also induce viral variability. There are even signs supported by theoretical considerations that some of these help HIV-1 to adapt its mutation rate to environmental requirements [7,8,9,10,11]. While a low mutation rate ensures viral integrity in a constant environment, a shift to an increased mutation rate ensures its adaptability in a changing environment. One mechanism to tune the HIV’s mutation rate is established through the interplay between the viral protein Vif (viral infectivity factor) and the host’s antiviral restriction factors of the APOBEC3 (apolipoprotein B mRNA-editing, enzyme-catalytic, polypeptide-like 3) family.

## 2. The Interplay between Host Restriction Factors from the APOBEC3 Protein Family and HIV-1 Vif in the Viral Replication Cycle

The survival of HIV-1 depends on specific interactions with cellular proteins that support or restrict its infection in human cells. Since the transfer of Simian immunodeficiency virus derived from chimpanzee (SIVcpz) to humans at the beginning of the twentieth century [12], viruses that evolved and formed different HIV-1 clades adapted to these human proteins, which include host restriction factors. The most prominent examples of HIV-1 restriction factors are APOBEC3G, TRIM5α, Tetherin and SAMHD1 [13,14]. These proteins are either constitutively expressed or induced by interferons and act in a non-secreted way directly to inhibit specific steps of the viral replication cycle in either virus producer or virus target cells. It appears that the main task of some HIV accessory proteins is to counteract cellular restriction factors. The viral Vif and Vpu proteins bind directly to APOBEC3G and Tetherin, respectively, and induce their degradation [13]. While in HIV-1 no protein evolved to inhibit SAMHD1, the related HIV-2 uses its Vpx protein to destroy this dNTPase [15,16,17].

### 2.1. APOBEC3

**Figure 1:**
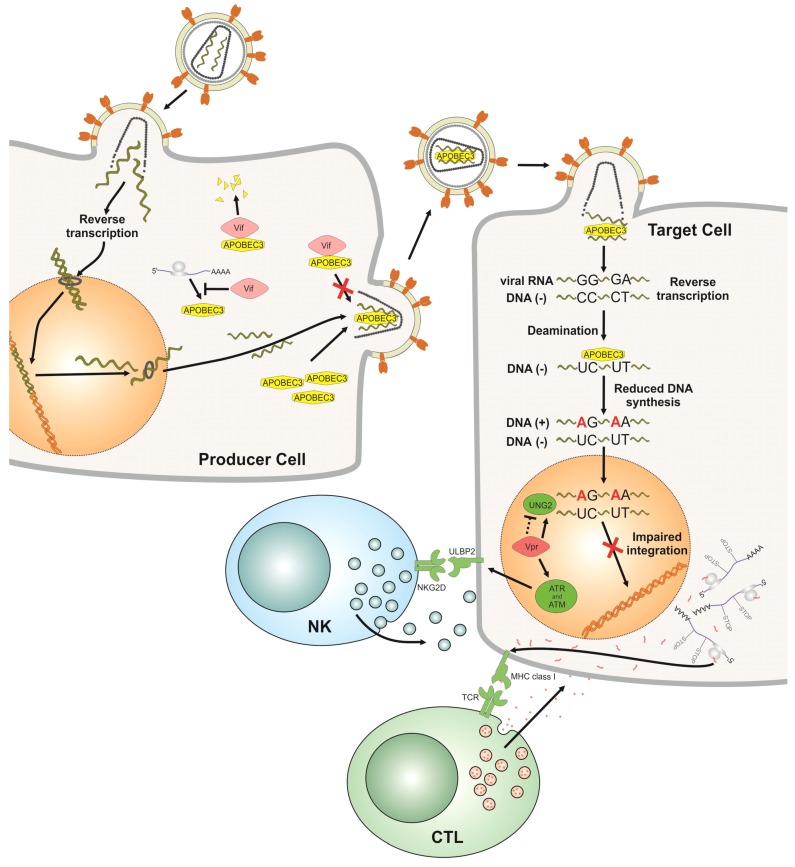
Impact of the cellular restriction factor APOBEC3 (A3) on human immunodeficiency virus-1 (HIV-1) replication. The HIV-1 replication cycle starts by infection of cells that express A3 proteins (producer cells). In the producer cells, viral infectivity factor (Vif) targets the A3 proteins for proteasomal degradation, but can also counteract the encapsidation of A3s by direct binding without degradation and by reducing the translation of A3 mRNA. If Vif is not expressed or does not bind to A3s, A3s are packaged into HIV-1 virions budding from the cells. During the next round of infection in target cells, encapsidated A3 proteins inhibit HIV-1. Single-stranded viral (−) DNA (generated by viral reverse transcription) serves as a substrate for A3-induced cytidine deamination, which causes G-to-A hypermutations in the viral (+) DNA. Additionally, the presence of A3 proteins inhibits reverse transcription and results in damaged ends in the double-stranded viral DNA. These inhibit integration. A3s also impair the integration of HIV-1 by binding directly to the integrase. The number of integrated, highly mutated proviruses is low. The base excision repair pathway replaces uracils with thymidines: Uracil-containing proviral DNA is first subject to removal of uracils by uracil DNA glycosylase (UNG) bound to Vpr. The damaged DNA activates the DNA sensors ATR and ATM, triggering the DNA damage response (DDR). The DDR can upregulate expression of ULBP2, a ligand for natural killer cell (NK) receptor NGK2D that sensitizes HIV‑infected cells to NK cell lysis. In addition, A3s improve the recognition of HIV‑infected cells by CD8^+^ cytotoxic T lymphocytes (CTL), as hypermutated proviruses encode mRNAs with missense and nonsense mutations that supply a pool of MHC-I-restricted HIV antigens. Killing of HIV-infected cells may result from a balance between these activating mechanisms and other viral pathways that repress cell recognition by NK cells and CTL.

HIV-1 is an RNA virus that infects cells by binding to CD4 and a chemokine co-receptor (mostly CCR5 or CXCR4). After the viral and cell membranes have fused and the viral core enters the cytoplasm, the viral RNA is reverse transcribed into double-stranded DNA by the viral reverse transcriptase. This viral DNA is integrated into chromosomal DNA by the viral integrase protein, generating a transcriptionally active provirus. Newly translated viral polyproteins assemble with viral genomic RNAs at the cytoplasmic membrane and, together with cellular proteins such as APOBEC3G, form nascent particles, which bud out of the infected cell (Figure 1).

APOBEC3G (A3G) was discovered in the effort to understand the lack of replication of HIV-1 lacking Vif gene expression (HIV-1ΔVif) in certain cells such as peripheral blood mononuclear cells (PBMCs) [18]. A3G belongs to the family of APOBECs that in humans includes AID, APOBEC1, APOBEC2, APOBEC4 and seven A3s (A3A–A3D and A3F–A3H). Cytidine deamination of single‑stranded DNA was shown to be the principal activity of the A3 proteins in biochemical and cell culture assays [19]. It appears that only placental mammals encode A3 genes, whose number is species-specific [20,21]. Depending on the experimental conditions, the infectivity of HIV-1ΔVif particles can be reduced by human A3B, A3F, A3D, A3G and A3H (haplotype II, hap II) proteins up to 1,000-fold by accumulative mechanisms. In contrast, A3A is inactive and A3C inhibits only very weakly [13,22]. In the target cells of HIV, A3A and A3B are barely detectable [23,24]. Under laboratory settings, Vif protein efficiently, but not completely, counteracts A3D, A3F, A3G and A3H [22]. 

Most knowledge of inhibition of HIV-1 through A3 proteins is derived from studies testing the A3F and A3G proteins. In HIV-1ΔVif infected cells, A3G can bind to the nucleocapsid (NC) part of the viral Gag polyprotein and is incorporated into the budding virus particle [13]. The presence of Vif protein in the cell prevents the packaging of Vif-sensitive A3 proteins such as A3F/G into nascent viruses. The interaction of A3G with the viral Gag protein and its subsequent incorporation into viral cores is required for A3G to inhibit the next round of infection. In the virion, A3G proteins are then processed and cleansed of an inhibitory RNA by the viral RNase H [25]. After cell entry, the viral genomic (+) strand RNA is reverse transcribed into (−) strand DNA that is the template for the (+) strand DNA synthesis, generating a complete, double-stranded DNA. These particle-delivered A3 proteins can inhibit HIV through multiple mechanisms early in the infection cycle, and viral genomes isolated in the first hours post infection contain many G-to-A mutations also called hypermutations [26,27,28,29]. A3 deaminates cytidines mainly on the viral (−) strand DNA mutating them to uracils (Figure 1). As a consequence, the viral coding (+) strand shows G-to-A changes. A3F and A3G prefer to deaminate cytosines in the dinucleotide contexts TC and CC (deaminated cytidine underlined), respectively. The frequency of the cytosine deaminations can be influenced by the amount of encapsidated A3 protein, by the specific type of A3 protein and the processivity of the reverse transcriptase [30]. The A3-induced editing of the viral genome can cause missense or nonsense mutations in viral genes and can damage the viral regulatory elements.

Antiviral cytidine deamination leaves uracil nucleobases in the HIV-1 cDNA. These can also arise by direct incorporation of dUTP during the reverse transcription process. Uracil is regularly only found in cellular RNA and not DNA. Uracil lesions in DNA are removed by uracil DNA glycosylases (e.g., UNG2, SMUG1) that initiate the base excision repair (BER) pathway. DNA molecules missing bases are then processed by apurinic/apyrimidinic endonuclease (APE) that nicks the DNA backbone, generating a 5'-deoxyribose phosphate group that is a substrate for DNA repair enzymes. It was initially hypothesized that UNG2 may contribute to the A3G-mediated loss of infectivity by generating abasic sites that would trigger degradation of the viral DNA or be a block to completion of reverse transcription [26]. While UNG2 has been additionally detected in HIV-1 particles, reports regarding the role of UNG2 in the viral infectivity of HIV-1ΔVif generated in A3G expressing cells and the role of the viral protein Vpr for UNG encapsidation have conﬂicting ﬁndings [31,32,33,34,35]. Yan *et al.* recently found that HIV cDNA formed in human primary cells is heavily uracilated, because the viral reverse transcriptase cannot distinguish between dTTP and dUTP [36]. This A3-independent uracilation is thought to promote the early stage of infection by preventing the disastrous auto-integration of viral DNA [36]. Together, the data show that (1) HIV-1 tolerates uracilation, and (2) a pro- or antiviral function for BER following natural dUTP incorporation or cytidine deamination by A3s appears less clear very early in the replication cycle. Once integration has occurred, it is presumed that uracils in proviral HIV DNA are efficiently removed and replaced by thymidines by UNG-triggered BER. Supportive recent data show that the removal of A3G-induced uracils in HIV-1 DNA by UNG2 (bound to Vpr) and APEs activates the DNA sensors ATR and/or ATM and generates a DNA-damage response (DDR) [37] (Figure 1). 

Very early after infection by HIV-1ΔVif, the amount of reverse transcribed viral DNA is two- to 26‑fold lower than normal due to the presence of encapsidated A3F and A3G proteins [27,28,35,38,39]. Several studies have found that A3F and A3G interfere with multiple steps of reverse transcription: The interaction of A3G with NC reduces the annealing of the tRNA primer [39,40,41], and A3G blocks the strand transfer steps [35,42] and further inhibits the elongation of HIV-1 DNA [43]. A steric hindrance of the reverse transcriptase could be caused by a direct interaction between A3G and the reverse transcriptase [44]. In addition, the presence of A3G results in the formation of aberrant 3' viral long terminal repeat (LTR) ends, suggesting that A3G also interferes with the cleavage and removal of the primer tRNA [35]. These damaged ends contribute to defects in plus-strand DNA transfer and cause inefficient integration. While A3G generates a 6 bp extension at the viral U5 end of the 3'LTR, A3F inhibits integration more potently by reducing the 3' processing of viral DNA by the integrase at both the U5 and U3 ends [39]. Furthermore, both A3G and A3F can directly interact with the integrase and negatively affect the integration efficiency of HIV-1Δ*vif* viruses by 5- to 50-fold [27,28,35,38,39].

While many of the studies cited above were performed with wild type A3 proteins, there is evidence that A3F and A3G mutants that lack cytidine deamination activity still demonstrate substantial anti‑HIV-1 activity and can inhibit the reverse transcription and integration of HIV-1ΔVif [38,39,41,42,43,45,46,47,48].

The HIV-1 proviral genomes with hypermutations may not be transcribed well, depending on whether mutations are present in the promoter regions or *tat* gene. Thus, it is likely that only moderately mutated genomes contribute to the gene pool of the viral population. Using a cell culture system with A3G and HIV-1ΔVif, Russel *et al.* found that the frequency of hypermutation was highest in viral DNA, reduced in cellular viral RNA, and lowest in virion RNA [49]. This gradient of hypermutation showed a purifying selection pressure against genomes that have inactivating mutations in the *gag* gene. In addition, transcribed viral RNAs with mutations may have a reduced stability and they may be degraded before they can be translated. Despite the purifying selection at multiple steps, viral genomes containing stop codons in *gag* were detected in released particles, indicating dual or multiple infections and complementation of the Gag defect [49]. This implies that G-to-A hypermutations can contribute to viral variation through recombination of co-packaged viral genomes.

Cytidine deamination might have additional immunological consequences for the patient as well (Figure 1). Primary CD4^+^ T cells infected with HIV-1ΔVif are more susceptible to lysis by Natural Killer (NK) cells than cells infected with wild-type (wt) HIV-1, because A3G activates the DDR by introducing uridines into DNA [37]. The ensuing DDR upregulates expression of the ligand for the NK cell receptor NGK2D on HIV-infected cells, sensitizing them to NK cell lysis. A3G is also reported to improve the CD8^+^ cytotoxic T lymphocyte (CTL) recognition of HIV-infected T cells [50]. CTL activation is enhanced by truncated or misfolded viral proteins expressed by A3G-edited viruses that supply a pool of MHC-I-restricted HIV antigens. Killing of HIV-infected cells by NK cells and CTL likely results from a balance among the efficacy of induction of DDR, generation of antigens enhanced by A3G-editing and the immune-escape mechanisms mediated by Vif and other viral proteins.

### 2.2. Vif

The Vif protein of HIV-1 is required for virus replication in A3F and A3G (A3F/G) expressing cell lines and primary cells [51,52,53,54,55]. Besides HIV, most other lentiviruses also encode *vif* genes. Vif binding to A3F/G will induce polyubiquitination and degradation of A3F/G by the cellular proteasomal machinery. The A3-Vif interaction is crititcal: A3 proteins that do not bind to Vif because the A3‑binding region is mutated or the particular A3 lacks a HIV-1 Vif-interaction surface are not counteracted and are likely to inhibit the virus. 

To achieve polyubiquitylation and subsequent degradation of A3F/G, Vif binds A3F/G and acts as an A3F/G substrate receptor molecule by mimicking the SOCS-box component of a cellular E3 ubiquitin ligase in the Vif-Cullin5-Elongin B/C ubiquitin ligase complex [56,57,58,59,60,61,62,63]. Vif additionally recruits the transcription cofactor CBF-β to form this active complex [64,65]. There is little knowledge about the regulation of Vif. It is not associated with kinases [66] and appears not to be a phosphor‑protein [67]. Several A3-Vif interaction motifs have recently been described in Vif such as for A3G the G-box (residues 40–44) together with the WxSLVK motif (residues 21–26), the FG-box (residues 55–72), the LGxGxxIxW motif (residues 81–89) and the T(Q/D/E)x_5_ADx_2_(I/L) motif (residues 96–107) [68,69,70,71,72,73]. Vif mutants that have a defect in binding to A3G, Cullin 5, or Elongin C are unable to counteract the antiviral activity of A3G by degradation. Besides degradation of A3G, Vif exploits other pathways to prevent packaging of A3G. In experimental systems where Vif-dependent A3G degradation was not seen, Vif still protected HIV-1 particles against A3G, suggesting that binding of Vif to A3G can be sufficient [74,75,76]. Thus it is possible that Vif induces structural changes in A3G that prevent packaging and/or inhibit the enzymatic function of A3G [77,78,79]. In addition, Vif may specifically also inhibit the translation of A3G mRNA by 30%–50% [80,81] 

## 3. Models for Viral Evolution and Host Pathogen Co-Evolution/Interactions

As a result of its short generation times and high mutation rates, HIV-1 evolves at short time scales of hours, days or months, allowing evolutionary trajectories to be followed in real time. Phylogenetics [82] can place observed viral sequences into the context of their evolutionary history through phylogenetic trees. These analyses give deeper insights into the realized evolutionary path, which, however, could have manifested quite differently in another occasion due to the random factors of the underlying evolutionary process. Mathematical models provide a means to go beyond the insights gained from a single evolutionary path and to assess the patterns and dynamics of evolution on the level of the viral population. Under the names of population genetics or quasi-species theory they describe the mutation-selection balance in self-replicating entities such as viruses [83,84]. Below, we review models of the impacts of mutation and selection on evolutionary dynamics such as adaptation to changing environments and co-evolution. A more fundamental understanding of evolutionary patterns found in the HIV Vif-A3 system can be achieved within these model frameworks.

### 3.1. Quasispecies in Static Environments

**Figure 2:**
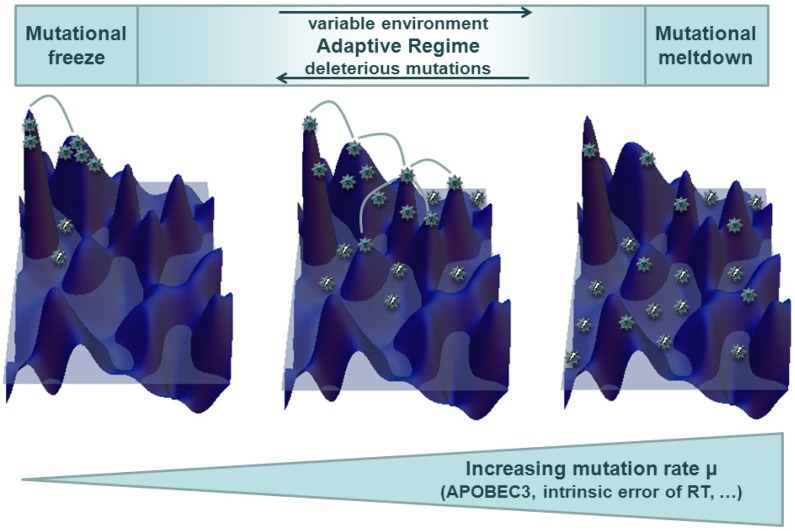
By increasing intrinsic mutation rates, the viral populations are able to explore larger areas of genomic sequence space. This facilitates better adaptation to variable environmental conditions and avoidance of mutational freeze. However, increased mutational load is accompanied by added deleterious mutations (due to deep valleys in the fitness landscape), which ultimately lead to mutational meltdown.

The concept of quasispecies was introduced by Eigen and Schuster [85,86] and describes the equilibrium distribution of viral mutants found under the mutation selection balance. It does not make specific assumptions about viral replication rates and the resulting fitness landscape [87]: In its simplest form it assumes that among genotypes of low replication competence there is only one strongly replication competent genotype—the so-called master sequence. The quasispecies can be thought of as a cloud of mutant sequences surrounding and including the fittest master sequence. The viral diversity in quasispecies increases up to a critical viral mutation rate, at which the quasispecies breaks down and the genomic information of the master sequence is lost in the cloud of mutant sequences (classical error catastrophe). This represents a breakdown of the mutation selection balance towards a regime in which the noise induced by mutations cannot be compensated for by the master sequence’s replication capacity. While viruses experience a more complex fitness landscape than a single peak fitness landscape (as sketched in Figure 2), this simplest realization of the quasispecies model remains instructive: It shows that mutations introduce couplings among viral sequences so that viral evolution can only be understood on the population level, or in terms of the quasispecies’ cloud of sequences. The simple approach further assumes that there is only a soft selection, meaning that the viral population has a constant size and cannot, by definition, become extinct. A first step from the single peak fitness landscape towards a more realistic fitness landscape is the introduction of deleterious mutations. Dynamics in this setting differ from the error catastrophe, in which a genotype cannot be sustained in a population due to mutational noise, and introduce an extinction threshold by lethal mutagenesis [88,89]. Hard selection will lead to a decrease in the viral population if the population’s mean fitness is too low, an event called mutational meltdown. The collective nature of the quasispecies becomes evident when moving from the simple single-peak fitness function towards more complex fitness landscapes. Increasing mutation rates may favor closely related sequences with moderate replication capacity over isolated sequences with high replication capacity, which are destabilized more easily by mutational losses. This is referred to as survival of the flattest [90,91].

Another assumption often made in the above approach is that the viral population is infinite, or at least large enough that every possible sequence can be found in representative proportions. The HIV genome’s length is about 10^4^ bases, each of which can be chosen from among four nucleotides and leads to 4^10,000^ ≈ 3.98 × 10^6,020^ potential mutant sequences. While the full combinatorial space of potential sequences is certainly not biologically relevant, it is still an unrealistic scenario to find a full representation of only a small proportion of these potential sequences in a viral population at any time. This does not invalidate the quasispecies theory, but shows that stochastic fluctuations become relevant and have to be taken into account [92,93,94]. Stochastic fluctuations manifest as genetic drift meaning that favorable mutations are not necessarily fixed on the population level [93]. In finite populations, deleterious mutations accumulate if a mutation originating from the master or consensus sequence is more likely than a back-mutation, as only very few of the possible mutants are present in minority populations. This successive accumulation of mutations is called Muller’s ratchet [92,95] and its rate increases with sequence length and decreases with population size. It has been shown that recombination (sexual reproduction) slows the rate of the ratchet [96], though recombination can exhibit quite a range of stabilizing as well as destabilizing effects [97,98,99,100,101,102].

The destabilizing effect of a high mutation rate is observed in a situation in which A3s are not sufficiently neutralized, due either to a defective or an absent HIV Vif protein. Experiments show that viral sequences are hypermutated to the extent of lethal mutagenesis [103,104,105], and modeling studies can help to explore the boundaries of the lethal regime for the virus and make predictions about viral viability [106,107].

### 3.2. Quasispecies in Dynamic Environments and Co-Evolution in the Host Context

Viruses face different conditions in each host as well as changes imposed by the host’s immune response [108]. An adequate model must take this into account. The simplest extension of a model with a quasispecies on a single peak-fitness landscape is to allow for the fitness peak to move at a certain rate to other (neighboring) sequences. This lowers the classical error threshold, but more importantly introduces another threshold that can be observed for small mutation rates: As soon as the mutation rate falls below a critical rate, the viral quasispecies experiences an “adaptation catastrophe” meaning that the quasispecies is no longer able to adapt to the changing environment [109]. Aside from the moving fitness peak, an oscillating fitness peak or landscape is another simple extension of a static fitness landscape in which the fitness peak’s height oscillates over time. It leads to a temporal phase shift between population size and fitness if adaptation lags behind the dynamic environment. In the case of even faster fitness oscillations, an averaged dynamic is observed [110,111]. While in static fitness landscapes, optimal mutation rates are constrained by the need to avoid deleterious mutations [112], survival of a quasispecies in dynamical fitness landscapes relies both on conservation of genomic information and adaptability, which puts other constraints on optimal mutation rates [113]. Figure 2 visualizes how mutation rates may adapt to the constraints in complex environments: Higher mutation rates are favored in variable environments, whereas a high probability for deleterious mutations requires higher replication fidelity. This is often studied for genomic mutation rates μ_G_ (instead of point mutation rates μ), which for genomes of length n is given by μ_G_ = 1 − (1 − μ)^n^ ≈ nμ. The optimal genomic mutation rate can be expressed by the ratio of point mutations to the next fitness peak and virus generations between peak shifts. This implies that most viruses will have accumulated the “right” number of point mutations when the fitness peak shifts [114,115]. 

Ultimately, viruses do not exist in an environment that changes independently from viral evolution. Adaptive immunity creates an environment that is itself shaped by the viral quasispecies. An adequate model has to consider these coevolutionary dynamics [116]. Taking the coevolutionary arms race into account introduces constraints on genome lengths and mutation rates, both for the virus [117,118] and for antigen-binding sites involved in an immune response, such as the complementarity determining regions of antibodies [119]. Viruses are most viable in the context of the coevolutionary model if their genomic mutation rate corresponds to the ratio of the viral generation time to the response time of the adaptive immune system. This implies that an escape mutant shows up in the viral population as soon as the immune system has caught up with the latest viral “master sequence”. The fact that viral generation time is the only viral parameter leaving its mark on the genomic mutation rate in this approximation is reminiscent of the fact that genomic mutation rates are of a similar order of magnitude among different classes of viruses [117]. These coevolutionary models have been further extended from single-stranded sequences to double-stranded DNA replication [120] as well as to finite viral populations [121]. Still they leave a demand for more realistic models of the complex interplay between viruses and adaptive immunity.

A3-induced hypermutation increases overall HIV mutation rates. This has been shown to increase viral diversity and to allow for faster adaptation to selective pressures [7,122], specifically the emergence of resistance towards therapeutic drugs [123,124].

## 4. Coevolution of HIV Vif and APOBEC3 — Impact on Viral Escape and Drug Resistance

### 4.1. *In Vitro* Evidence of HIV Vif APOBEC3 Coevolution

HIV-1’s ability to replicate in the presence of A3F/G strictly relies on the Vif protein as described above. More than 20 years ago, it was observed that G-to-A hypermutations in viral genomes are found in cultures of primary HIV-1 [125]. Vif variants defective in binding either A3F or A3G can be detected in HIV-1 patients, implying that the Vif-mediated counteraction of A3 is not absolute [8]. Here, the identified Vif mutations K22E, S32P, Y40H, E45G, F115S, G138R, or L150P did not restore the HIV-1 infectivity in the presence of A3G [8]. *In vitro* experiments using HIV-1 with either Vif.K22E or E45G revealed increased A3G-mediated cytidine deamination in viral genomes that induced the appearance of M184I mutant viruses resistant to the antiviral drug lamiduvine (3TC) before drug exposure [123,126]. The incomplete A3 neutralization increased the genetic diversity and the mutation frequency inversely correlated with the fitness of HIV-1 Vif mutants in PBMCs [123]. This study also showed that hypermutated HIV-1 generated replication-competent drug resistant viruses only through recombination with wt HIV-1 [123]. In other experiments, even wt HIV-1 could develop resistance to lamiduvine by acquiring the M184I mutation in cells expressing A3G faster than in A3G-non-expressing T cells [122].

### 4.2. *In Vivo* Clues of HIV Vif APOBEC3 Co-Evolution: Clinical Data on HIV Dynamics in Patients

All exogenous lentiviruses, except the equine infectious anemia virus, rely on a Vif protein for productive infection of their host, showing that Vif is essential *in vivo* [127,128,129,130,131]. In wt Simian immunodeficiency virus (SIV) infected Rhesus macaques, it was shown that increased A3F/G expression is associated with low viral load and prolonged survival of the infected monkeys [132]. However, the interplay between HIV Vif and A3 appears to be more complex in the human host. There is also considerable variation during the disease, specifically regarding the time from first exposure to HIV-1 to the development of AIDS. Some of the long-term non-progressing individuals, elite‑controllers and controllers, remain free from AIDS because of very low virus loads suggesting a situation similar to that observed in Rhesus macaques infected with SIV. In contrast, HIV-1 sequences carrying the footprints of A3-induced mutations have been isolated in treated and untreated chronically and early-infected patients [133,134,135,136,137,138,139], infected infants [140,141,142], and elite controllers [8,143]. Studies that looked for a correlation of the extent of hypermutation in HIV-1 and clinical markers (viremia, CD4^+^ cell numbers) in infected patients resulted in contradictory findings [133,142,144,145,146,147,148]. Some studies found an inverse correlation between A3 expression and viral load [145,149,150,151], while others found no such correlation [141,152,153]. HIV-negative individuals reportedly have higher A3G expression compared to most HIV-1 patients, suggesting that A3G transcription is rapidly down regulated upon HIV-1 infection [152,153]. In contrast, it was reported that chronically infected untreated patients expressed higher levels of A3G than healthy control individuals and patients under antiretroviral therapy [151,154]. *In vitro* findings show that expression of A3G is upregulated upon CD4^+^ T cell activation [23] and A3G expression during HIV-1 infection may decrease over the course of disease progression [151].

In addressing the potential association between the degree of hypermutation and disease progression attempts have been made to identify specific relevant mutations in the *Vif* and A3 proteins. The *vif* gene is not excluded from HIV-1 diversification and natural variations in Vif are frequently identified [8,133,155,156,157,158,159,160,161]. With the current knowledge, it is difficult to speculate about the way in which these particular Vif variants might contribute to viral pathogenicity. Vif variants could have an increased capacity to counteract A3 proteins or a diminished anti-A3 activity. Higher Vif activity would reduce the residual inhibition by A3s, but also decrease the sublethal level of A3 editing that might facilitate the emergence of immune escape or drug resistant viral forms. Thus, an accelerated disease progression may be determined by an increase or a decrease of Vif activity. Genetic polymorphisms in proteins of the E3 ubiquitin ligase complex such as Cullin 5 might be an important additional factor regulating the Vif-induced degradation of A3s [142,162].

Vif proteins derived from different HIV-1 subtypes of group M do not all show the same defense activity against the reference A3F, A3G and A3H hapII [9,158,163]. In one study, subtype C-derived Vif proteins had the highest activity against A3G [9] in agreement with the observation that G-to-A hypermutations in viral genomes are less frequently detectable in patients infected with subtype C viruses than with patients infected with other subtypes [135]. In contrast, HIV-1 group M subtype Vifs show a similar activity against A3F and A3G, but differ in their capacity to counteract A3H hapII, and only subtype F Vifs are highly effective in inhibiting A3H hapII [163]. Interestingly, the allele frequency of the active A3H hapII differs considerably among human ethnicities, being high in African and low in European and Asian populations [164,165]. Since patient-derived A3s and Vif-interacting proteins were not tested until recently, it is unclear whether the subtype Vifs show an adaption to human genetic variability.

*In vitro* studies suggest that the genetic reservoir necessary for viral escape from particular antiretroviral inhibitors and inhibitory immune responses is enhanced in HIV-1 strains encoding partially active Vif alleles. Viruses with known Vif mutations in A3-binding sites can be detected *in vivo* [7,8,123,133] and might be more frequent in patients failing antiretroviral therapy suggesting that sublethal editing is a source of drug resistance mutations [126]. Hassaïne *et al.* found that the amino acid at position 132 of Vif is associated with low viral load in HIV-infected long-term non‑progressing individuals [166]. Other studies on long-term non-progressors failed to confirm the presence of specific Vif variants in most patients [167,168]. However, in single cases, severely defective Vif variants may contribute to non-progression to AIDS [169,170]. Accelerated progression to AIDS also was found to be associated with Vif in a pediatric HIV-1 cohort: Insertion of one amino acid (an alanine or a threonine residue) at position 61 (INS61), and substitutions A62D/N/S and Q136P were individually associated with accelerated disease progression [171]. Positions 61 and 62 are located within a conserved A3F/G interaction motif [70], and Q136P is in the conserved zinc-binding HCCH box of Vif. 

Searching for factors involved in HIV-1 permissiveness or disease progression, several groups also looked for correlations with mutations in A3G. While these studies identified many polymorphisms in A3G, most of the SNPs did not associate with increased risk of infection, rate of disease progression or hypermutation of viral genomes [133,153,171,172,173,174,175]. Four exceptions are known. The first, the H186R (rs8177832) mutation, a codon-changing variant in exon 4, is relevant in African Americans but not in Caucasians. The second is the 5' extragenic mutation (rs5757463). The third and fourth are the a 3' extragenic mutation (rs35228531) and C40693T (rs17496018) in intron 4, and were found to be associated with high viral loads and decreased CD4^+^ T cell levels or increased risk of infection, respectively [153,173,175,176]. Some A3G polymorphisms might not affect disease progression, because HIV-1 adapts and evolves specific Vif variants, as described in a pediatric cohort where Vif.E45D associated with the A3G C40693T allele [171]. It was also reported that A3H may cause a detectable mutational signature on HIV-1 genomes *in vivo * [172]. That study identified three A3H SNPs in HIV-1 patients, linked to the previously described, less active A3H haplotypes [164,177] with reduced G-to-A mutations in viral DNA and a lower HIV-1 RNA level. The authors speculate that lower A3H activity may slow HIV-1 sequence diversiﬁcation and escape from immune responses, leading to lowered viral loads [172]. These data suggest that A3H proteins play an important role in HIV-1 sequence diversiﬁcation and evolution. 

**Figure 3:**
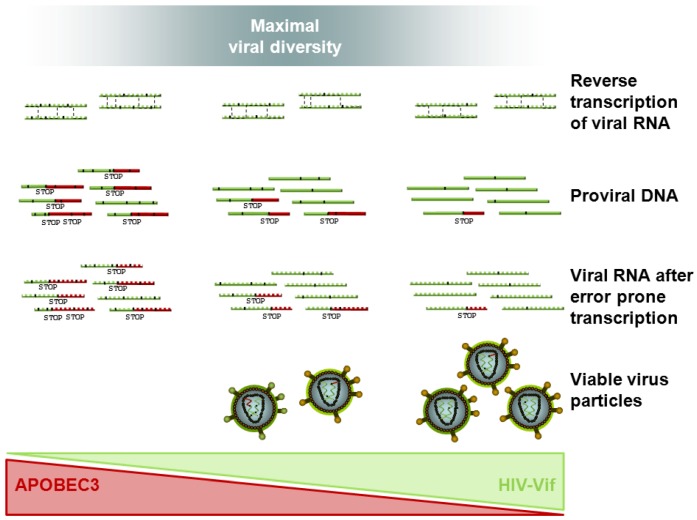
The APOBEC3-HIV-1 Vif balance determines the viral mutation rate in particular with respect to G-to-A hypermutations. The higher the mutation rate (right to left) the more mutations (black marks) are introduced into the viral genome during the viral replication cycle, including nonsense mutations (STOP codons, represented by a following red genome sequence) and missense mutations leading to a change in the coded amino acid. Recombination by template switching during reverse transcription (sketched by a black dashed line) is likely to alleviate the deleterious effect of mutations and to allow for higher viral variability. Increasing the mutation rate decreases the number of viable virus particles but drives the viral population through a regime of maximal diversity before extinction occurs through lethal mutagenesis (right to left).

Considering the complex interplay between mutation rates and environmental conditions in viral evolution (as discussed in Section 3), it is not too surprising that no simple dependency has been derived so far between clinical disease progression and viral mutation rate as expressed by the A3-Vif balance. Figure 3 sketches how the A3-Vif balance may influence viral diversity and the amount of viable virus particles in a complex and environment dependent manner: A virus is best adapted to a constant environment (weak immune response) through a low mutation rate, avoiding mutational losses and achieving a high viral load. In a changing environment shaped by a strong immune response, the viral population will be better off trading the viral load for higher diversity and the option of regular escape. These features will not be easily accessible by simple correlation studies but will require more advanced modeling than is currently available.

### 4.3. Tracing HIV Vif-APOBEC3 Coevolution in the Genome — Towards an Interpretation and Extrapolation of *in Vitro* and *in Vivo* Data through Modeling Studies

HIV-1 displays a continuous range of hypermutation rates [178], and A3-mutated viral populations appear to be more adaptive to selective pressures, including the emergence of drug resistance [8,122,123,124] and expansion of the Env receptor interaction [179,180]. These facts suggest that HIV might be able to tune its mutation rate through its Vif protein [22,181]: That is, if HIV profits from increased mutation rates in variable or challenging environments, increased rates might be selected for through the Vif protein [7,8]. While this qualitative picture is plausible, modeling approaches have not yet consistently linked viral hypermutation to disease progression. Instead, rather specific conclusions have been drawn from data analyses and modeling studies. These often appear contradictory if not distinguished with respect to the very specific questions they ask or interpretations they make. As A3 induces mutations from guanine (G) to adenine (A) biases in these nucleotides are often considered to be fingerprints of evolution under the influence of A3. These biases are evaluated against null models, which are often supplemented by computer simulations. 

The observation that adenine is preferred over guanine in retroviral codon usage might suggest that this has emerged in response to A3 pressure. However, in sequence positions in which A and G are synonymous choices, their occurrence is not correlated with the presence or absence of A3G or HIV Vif, suggesting other mechanisms underlying this bias towards A for example, biases in reverse transcription [182]. 

Other approaches looking at G *vs.* A biases also consider the motifs preferred by A3 in the larger sequence context of the editing site as well as the underlying evolutionary process [183]. The authors argue that synonymous G-to-A mutations are more likely to become fixed than nonsynonymous mostly deleterious ones. They consider adenine in an A3-preferred sequence context as putative ancestral guanines and test whether this hypothetical ancestral G-to-A mutation has been synonymous. They hypothesize that purifying selection should have the effect that current guanines in the A3 context are the “remaining guanines” deprived of synonymous mutations towards A. In other words, current guanines in the A3 context are less likely to mutate synonymously to adenine than was observed in the putative ancestral context, which the authors interpret as an A3 footprint. A more recent publication could not reproduce these findings in a generalized framework. In this later study, the authors could neither detect biases in A3-preferred sequence motifs towards the mutated versions nor the hypothesized ancestral changes in synonymous *vs.* nonsynonymous mutations [184]. However, the model does not take into account the collective impact of successive mutations by different A3 variants. Considering further that one of the preferred A3F/G target motifs, TGG is converted to a stop codon TAG under A3 hypermutation, A3F/G might lead to mostly lethal hypermutants leaving only a minor contribution of hypermutants to be found in the viable virus pool. 

A comprehensive model of the interactions between proteins of the A3 family and HIV Vif also relies on the knowledge of A3 packaging as well as of editing mechanisms and their efficiency in the target cells [185]. Experimental studies show that A3G incorporation into target cells is proportional to the expression of the protein [186] and a successive decrease in virus infectivity [105]. At the same time there is evidence that the incorporation of only a single or few A3G proteins per virion leads to a strong (or even lethal) reduction in viral replication and infectivity [186,187]. A combined *in vitro* and *in silico* approach points in a similar direction: It follows the probability of sequences to be hypermutated through a titration experiment with increasing availability of wt A3G. Using a maximum likelihood approach, the maximal number and distribution in the number of A3 proteins incorporated in the HIV virions was estimated. Even in viruses that were estimated to have only one A3G protein incorporated, mutation rates were observed that lead *in vitro* and *in silico* to mostly lethal offspring sequences with stop codons [188]. Considering the evidence of a continuous range of observed hypermutation rates, there must be a mechanism to “rescue” these mutated sequences. This suggests that HIV replication capacity can compensate for remarkable levels of mutational losses and/or that other evolutionary processes such as recombination are essential to ensure viral recovery and escape from A3 activity. Indeed, there is evidence of strong purifying selection during the viral replication cycle, leading to a reduction of the level of mutations from DNA via cellular viral RNA to virus RNA [49]. Recombination occurs in HIV, as the reverse transcriptase switches templates about 2–13 times per genome replication [189,190,191]. The combined effect of mutation and recombination and purifying selection on viral evolution is sketched in Figure 3: Frequent G-to-A mutations increase viral diversity, but also introduce early stop codons. Recombination can be a means for the virus to profit from its diverse repertoire while at least partially evading the deleterious effects of stop codons. The effect of recombination on emergence of drug resistance mutations has been studied [192,193,194,195,196,197] and its relevance is worth further investigation in the context of hypermutation. 

It has also been hypothesized that a mechanism similar to the one observed for the emergence of drug resistance might be a driver in the HIV-1 co-receptor switch. During the course and progression of disease, HIV often changes its use of a co-receptor for cell entry from CCR5 to CXCR4, consistent with a shift from G- to A-containing codon triplets [179]. The authors equally acknowledge the substantial impact of reverse transcriptase, which creates enough diversity to facilitate a co-receptor switch. As for the emergence of drug resistance, future research should reveal whether the increased A3-induced mutational load stabilizes minority populations that otherwise might become extinct due to stochastic fluctuations. In consequence, resistance mutations or a co-receptor switch could be established more rapidly and more easily. 

Another mathematical model, based on ordinary differential equations, has been used to study the intra- and intercellular kinetics of HIV replication. The model specifically considers the interaction between A3 and Vif and allows for simulations of viral production in different cellular contexts and under therapy regimes, though it does not take into account the impact of hypermutation on viral evolution (escape *vs.* mutational meltdown) [198].

There is still a need for a consistent understanding of the co-evolution of A3 and HIV-1 Vif, which already has been studied in the analogous system for SIV within subspecies of African green monkeys [10]. Vif has shown a remarkable adaptability to changing environments, and this could be a mechanism to tune viral mutation rates in the human host. A consistent modeling framework could provide insights into the relevant mechanisms and promote a less ambiguous interpretation of nucleotide biases in the HIV genome. In order to cover relevant evolutionary mechanisms, a model should consider the implications of the complex and variable fitness landscape HIV faces in its host [199,200,201,202,203,204]. This is in particularly challenging, as the fitness landscape seen by the virus responds to viral evolution through a coevolution facilitated by adaptive immunity. In addition, the finite size of the viral population further enhanced through compartmentalization introduces stochastic fluctuations that become especially relevant for the emergence of escape or resistant mutant populations.

## 5. Conclusions

The question of an optimal antiviral strategy can only be answered in a context-dependent manner. This leaves questions of clinical relevance open for future research: Should Vif be antagonized or aided in an anti-retroviral therapy v2.0 that uses the Vif-A3 interaction as a novel target? Does the virus replication *in vivo* depend on an optimal mutation rate and what is optimal under which conditions?

New tools to manipulate Vif and A3G have been developed and tested in experimental cell culture systems, but not yet applied in clinical studies. It seems obvious that if a treatment could inhibit the viral Vif protein, the cellular A3 proteins would be free to kill the virus, and much research is guided by this idea. Small inhibitory compounds were identified that bind to Vif or A3G and prevent their interaction or the Vif interaction with the E3 ubiquitin ligase complex protein Elongin C (see Section 2) to stabilize A3G [205,206,207,208]. Several groups explored molecules that could be used for gene therapy and showed that A3G that are fused to either the viral proteins Vpr (Vpr14-88.A3G) or a non‑pathogenic Nef variant (Nef7.A3G) escape the Vif-mediated degradation and efficiently inhibit wt HIV-1, presumably because the fused protein impairs the A3G-Vif interaction [209,210,211]. Similarly, Vif-resistant A3A that is not targeting the viral nucleoprotein complex and thus does not inhibit HIV-1 wt or HIV-1ΔVif, can be made an antiviral protein by fusion to Vpr (Vpr.A3A) or the N-terminal half of A3G (A3G_NT_.A3A), which deliver A3A to viral cores [212,213]. An opposite strategy aimed at reducing mutation rates was followed by identifying a small molecule inhibitor for A3G [214]. The authors speculate that inhibition of A3G could reduce the sublethal editing of HIV-1 by A3G and reduce quasispecies variability, making the virus more susceptible to control by the adaptive immune system and reducing drug resistance. 

Whether an increase or decrease in viral mutation rates is favorable to the viral quasispecies depends on its current mutation rate and its environmental challenges, the extent to which mutations are deleterious (the structure and ruggedness of the fitness landscape), the replication rate and the strength of purifying selection or the variability of the environmental conditions (immune response, antiretroviral therapy). Modeling studies can help to integrate these factors into a consistent framework relating to empirical observations. Consideration of the complex dynamics of virus-host interaction can provide an interpretation of often ambiguous or seemingly contradictory empirical observations. 
